# Trimethyl 2,2′,2′′-[1,3,5-triazine-2,4,6-tri­yltris­(aza­nedi­yl)]triacetate

**DOI:** 10.1107/S1600536810047604

**Published:** 2010-11-20

**Authors:** Sérgio M. F. Vilela, Filipe A. Almeida Paz, João P. C. Tomé, Verónica de Zea Bermudez, José A. S. Cavaleiro, João Rocha

**Affiliations:** a Department of Chemistry, University of Aveiro, CICECO, 3810-193 Aveiro, Portugal; b Department of Chemistry, QOPNA, University of Aveiro, 3810-193 Aveiro, Portugal; c Department of Chemistry, CQ-VR, University of Trás-os-Montes e Alto Douro, 2500-801 Vila Real, Portugal

## Abstract

The title compound, C_12_H_18_N_6_O_6_, was synthesized *via* nucleophilic substitution by reacting 2,4,6-trichloro-1,3,5-triazine with glycine methyl ester hydro­chloride in reflux (dried toluene) under anhydrous atmosphere. Individual mol­ecules self-assemble *via* strong N—H⋯O hydrogen bonds into supra­molecular double tapes running parallel to the [010] crystallographic direction. The close packing of supra­molecular tapes is mediated by geometrical reasons in tandem with a number of weaker N—H⋯O and C—H⋯N hydrogen-bonding inter­actions.

## Related literature

For background to nucleophilic reactions of 1,3,5-triazine, see: Blotny (2006 ▶); Giacomelli *et al.* (2004 ▶). For coordination polymers based on *N*,*N′*,*N′′*-1,3,5-triazine-2,4,6-triyltrisglycine, see: Wang *et al.* (2007*a* ▶,*b* ▶,*c* ▶). For previous work from our research group on the synthesis of derivatives of 2,4,6-trichloro-1,3,5-triazine from reactions with glycine methyl ester hydro­chloride, see: Vilela *et al.* (2009**a* ▶,b* ▶).
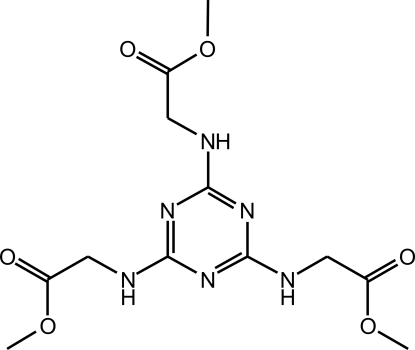


## Experimental

### 

#### Crystal data

C_12_H_18_N_6_O_6_
*M* *_r_* = 342.32
Monoclinic, 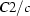

*a* = 24.0808 (11) Å
*b* = 9.4111 (4) Å
*c* = 15.5791 (7) Å
β = 116.018 (3)°
*V* = 3172.8 (3) Å^3^
*Z* = 8
Mo *K*α radiation
μ = 0.12 mm^−1^
*T* = 150 K
0.19 × 0.16 × 0.06 mm

#### Data collection

Bruker X8 Kappa CCD APEXII diffractometer
Absorption correction: multi-scan (*SADABS*; Sheldrick, 1998) ▶ *T*_min_ = 0.978, *T*_max_ = 0.993
27764 measured reflections
4182 independent reflections
2794 reflections with *I* > 2σ(*I*)
*R*_int_ = 0.043

#### Refinement

*R*[*F* ^2^ > 2σ(*F* ^2^)] = 0.042
*wR*(*F* ^2^) = 0.112
*S* = 1.03
4182 reflections
229 parameters
3 restraints
H atoms treated by a mixture of independent and constrained refinement
Δρ_max_ = 0.24 e Å^−3^
Δρ_min_ = −0.23 e Å^−3^

### 

Data collection: *APEX2* (Bruker, 2006 ▶); cell refinement: *SAINT-Plus* (Bruker, 2005 ▶); data reduction: *SAINT-Plus*; program(s) used to solve structure: *SHELXTL* (Sheldrick, 2008 ▶); program(s) used to refine structure: *SHELXTL*; molecular graphics: *DIAMOND* (Brandenburg, 2009 ▶); software used to prepare material for publication: *SHELXTL*.

## Supplementary Material

Crystal structure: contains datablocks global, I. DOI: 10.1107/S1600536810047604/gk2317sup1.cif

Structure factors: contains datablocks I. DOI: 10.1107/S1600536810047604/gk2317Isup2.hkl

Additional supplementary materials:  crystallographic information; 3D view; checkCIF report

## Figures and Tables

**Table 1:** Hydrogen-bond geometry (Å, °)

*D*—H⋯*A*	*D*—H	H⋯*A*	*D*⋯*A*	*D*—H⋯*A*
N4—H4⋯O4^i^	0.93 (1)	2.07 (1)	2.9851 (16)	168 (2)
N5—H5⋯O4^ii^	0.94 (1)	1.97 (1)	2.9097 (16)	172 (2)
N6—H6⋯O2^iii^	0.94 (1)	2.29 (1)	3.0733 (17)	141 (1)
C9—H9*C*⋯N1^i^	0.98	2.62	3.546 (3)	158

Symmetry codes: (i) 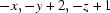; (ii) 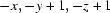; (iii) 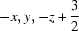.

**Fig. 1.**
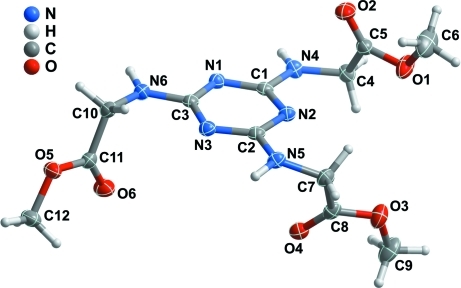
Schematic representation of the molecular unit of the title compound. Non-hydrogen atoms are represented as thermal ellipsoids drawn at the 50% probability level and hydrogen atoms as small spheres with arbitrary radii. The atomic labeling scheme is provided for all non-hydrogen atoms.

**Fig. 2.**
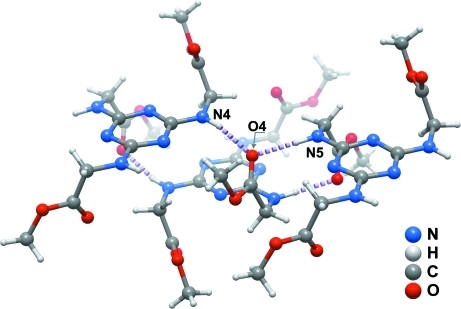
Fragment of the crystal structure emphasizing the contacts interconnecting adjacent chemical entities. The C=O4 carbonyl groups act as double acceptors in strong and highly directional N—H···O hydrogen bonds promoting the formation of a supramolecular double tape. For geometric details on the represented hydrogen bonds see Table 1. Symmetry transformations used to generate equivalent atoms have been omitted for clarity.

**Fig. 3.**
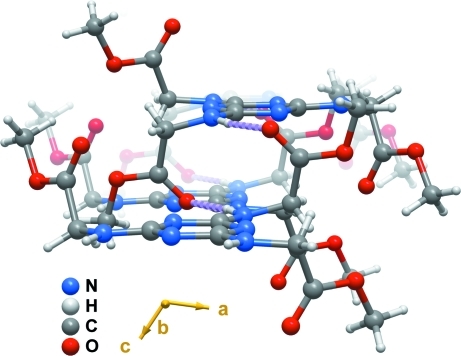
Supramolecular double tape formed by strong and highly directional N—H···O hydrogen bonding interactions viewed in perspective along the [010] direction of the unit cell.

**Fig. 4.**
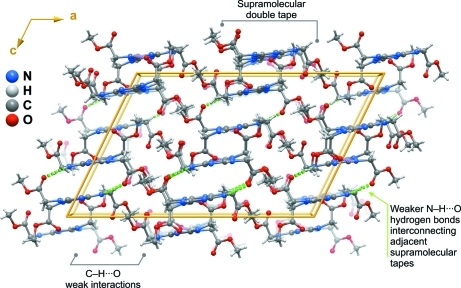
Crystal packing viewed in perspective along the [010] direction of the unit cell. N—H···O hydrogen bonds are represented as purple (intra-tape) or green (inter-tape) dashed lines. For hydrogen bonding geometric details see Table 1.

## supplementary crystallographic information

### Comment

2,4,6-Trichloro-1,3,5-triazine is a versatile organic molecule which can be used for the design and construction of larger entities because the three chlorine atoms are prone to nucleophilic substitution by several functional groups to form amides, nitriles and carboxylic acids, among several others (Blotny, 2006; Giacomelli *et al.*, 2004). Resulting compounds exhibit specific physico-chemical properties which render them of potential academic and industrial interest (*e. g.*, in the textile and pharmaceutical industries). Following our interest on crystal engineering of functional solids we have been using 2,4,6-trichloro-1,3,5-triazine as a molecular canvas for the design and synthesis of novel multipodal organic ligands. For instance, we have recently reported the synthesis and structural characterization of the monosubstituted form of the title compound: methyl 2-(4,6-dichloro-1,3,5-triazin-2-ylamino)acetate (Vilela *et al.*, 2009*a*). Following the same reaction procedure we were able to isolate the title compound (the trisubstituted derivative) as a pure phase. Noteworthy, the title molecule can be a precursor of *N*,*N'*,*N''*-1,3,5-triazine-2,4,6-triyltrisglycine which has been used in the construction of a number of transition metal coordination polymers (Wang *et al.*, 2007*a*,*b*,*c*).

The complete nucleophilic substitution of the chlorine atoms of the chlorotriazine ring by methyl glycinate (Vilela *et al.*, 2009*b*) led to the isolation in the solid state of the title compound (see Scheme). This novel compound crystallizes in the monoclinic centrosymmetric *C*2/c space group with one whole molecular unit composing the asymmetric unit as represented in Figure 1. The presence of three pendant substituent groups imposes significant steric impediment around the aromatic ring, ultimately preventing the existence of onset π-π stacking interactions as reported in the crystal packing of the monosubstituted analogue compound (Vilela *et al.*, 2009*a*). In addition, the spatial arrangement of the pendant groups promotes a minimization of the overall steric repulsion: adjacent pendant moieties are either pointing toward different sides of the ring or, when located on the same side, the N—C bond rotates so the groups are as far away as possible from each other (Figure 1).

The N—H groups are hydrogen-bonded to two carbonyl groups from adjacent molecular units. O4 acts as a double acceptor of two strong (*d*_D···A_ in the 2.91–2.99 Å range) and highly directional [<(DHA) angle in the 168–172° range] N—H···O hydrogen bonding interactions (Figure 2 and Table 1). These interactions lead to the formation of a double tape of molecular units running along the [010] crystallographic direction. As represented in Figure 3, the pendant groups point outwards of the double tape, thus allowing for an effective close packing of tapes in the crystal structure. Besides these pure geometrical reasons, the N6—H6 moieties located in the periphery establish physical connections between adjacent supramolecular tapes *via* a weaker N—H···O hydrogen bond (not shown; see Table 1 for geometrical details). It is also worth to mention that the presence of several crystallographically independent —CH_2_— and terminal —CH_3_ groups in close proximity with nitrogen and oxygen atoms promotes the existence of several weak C—H···(N,O) contacts which further strengthen the connections between adjacent molecular units (not shown). This structural feature is particularly important in the parallel close packing of tapes along the [100] direction (Figure 4).

### Experimental

Glycine methyl ester hydrochloride (193 mg, 2.17 mmol; Sigma-Adrich, 99%) and potassium carbonate (200 mg, 1.45 mmol; Sigma-Aldrich, >99.0%) were added at 273 K to a solution of 2,4,6-trichloro-1,3,5-triazine (100 mg, 0.542 mmol; Sigma-Aldrich, >98,0%) in dried toluene (*ca* 5 ml). The reaction mixture was kept under magnetic stirring and slowly heated to reflux under anhydrous atmosphere. The progress of the reaction was monitored by TLC and stopped after 24 h. The reaction mixture was then separated by flash column chromatography using as eluent a gradient (from 0 to 5%) of methanol in dichloromethane. The third isolated fraction was identified as the title compound (27% yield). Single crystals suitable for X-ray analysis were isolated from recrystallization of the crude product from a solution of dichloromethane: methanol (*ca* 1: 1). All employed solvents were of analytical grade and purchased from commercial sources.

^1^H NMR (300.13 MHz, CDCl_3_/DMSO-*d_6_*) δ: 3.45 (*s*, 9H, OCH_3_), 3.74–3.86 (*m*, 6H, CH_2_), 5.64–5.81 (*m*, 3H, NH). ^13^C NMR (75.47 MHz, CDCl_3_/DMSO-*d_6_*) δ: 41.9 (CH_2_), 51.4 (OCH_3_), 165.3 (CNH), 170.7 (CO_2_Me). MS (TOF MS ES+)*m/z*: 343.1 (*M*+H)^+^. Selected FT—IR data (ATR, in cm^-1^): ν(N—H) = 3347*m*; ν*_asym_*(—CH_3_) = 2957*m*; ν(C=O) = 1725*vs*; ν*_in-plane_*(ring) = 1493*s* and 1515*s*; δ(—CH_3_) = 1407*m*; ν(C=N) = 1370*m*; ν*_asym_*(C—O—C) = 1206*s*; ν*_sym_*(C—O—C) = 1165*s*; γ(ring) = 841*s*.

### Refinement

Hydrogen atoms bound to carbon were located at their idealized positions and were included in the final structural model in riding-motion approximation with C—H distances of 0.99 Å (—CH_2_— groups) or 0.98 Å (terminal —CH_3_ groups). The isotropic thermal displacement parameters for these atoms were fixed at 1.2 (—CH_2_—) or 1.5 (—CH_3_ moieties) times *U*_eq_ of the carbon atom to which they are attached.

All hydrogen atoms associated with the NH moieties were directly located from difference Fourier maps and included in the structure with the N—H distances restrained to 0.95 (1) Å and with *U*_iso_ fixed at 1.5 times *U*_eq_ of the N atom to which they are attached.

### Crystal data

**Table:**

C_12_H_18_N_6_O_6_	*F*(000) = 1440
*M**_r_* = 342.32	*D*_x_ = 1.433 Mg m^−^^3^
Monoclinic, *C*2/*c*	Mo *K*α radiation, λ = 0.71073 Å
Hall symbol: -C 2yc	Cell parameters from 5859 reflections
*a* = 24.0808 (11) Å	θ = 2.4–27.5°
*b* = 9.4111 (4) Å	µ = 0.12 mm^−^^1^
*c* = 15.5791 (7) Å	*T* = 150 K
β = 116.018 (3)°	Plate, colourless
*V* = 3172.8 (3) Å^3^	0.19 × 0.16 × 0.06 mm
*Z* = 8	

### Data collection

**Table:**

Bruker X8 Kappa CCD APEXII diffractometer	4182 independent reflections
Radiation source: fine-focus sealed tube	2794 reflections with *I* > 2σ(*I*)
graphite	*R*_int_ = 0.043
ω and φ scans	θ_max_ = 29.1°, θ_min_ = 3.6°
Absorption correction: multi-scan (*SADABS*; Sheldrick, 1998)	*h* = −32→32
*T*_min_ = 0.978, *T*_max_ = 0.993	*k* = −12→12
27764 measured reflections	*l* = −21→21

### Refinement

**Table:**

Refinement on *F*^2^	Primary atom site location: structure-invariant direct methods
Least-squares matrix: full	Secondary atom site location: difference Fourier map
*R*[*F*^2^ > 2σ(*F*^2^)] = 0.042	Hydrogen site location: inferred from neighbouring sites
*wR*(*F*^2^) = 0.112	H atoms treated by a mixture of independent and constrained refinement
*S* = 1.03	*w* = 1/[σ^2^(*F*_o_^2^) + (0.0517*P*)^2^ + 1.3693*P*] where *P* = (*F*_o_^2^ + 2*F*_c_^2^)/3
4182 reflections	(Δ/σ)_max_ < 0.001
229 parameters	Δρ_max_ = 0.24 e Å^−^^3^
3 restraints	Δρ_min_ = −0.23 e Å^−^^3^

### Special details

**Table:**

Geometry. All e.s.d.'s (except the e.s.d. in the dihedral angle between two l.s. planes) are estimated using the full covariance matrix. The cell e.s.d.'s are taken into account individually in the estimation of e.s.d.'s in distances, angles and torsion angles; correlations between e.s.d.'s in cell parameters are only used when they are defined by crystal symmetry. An approximate (isotropic) treatment of cell e.s.d.'s is used for estimating e.s.d.'s involving l.s. planes.
Refinement. Refinement of *F*^2^ against ALL reflections. The weighted *R*-factor *wR* and goodness of fit *S* are based on *F*^2^, conventional *R*-factors *R* are based on *F*, with *F* set to zero for negative *F*^2^. The threshold expression of *F*^2^ > σ(*F*^2^) is used only for calculating *R*-factors(gt) *etc*. and is not relevant to the choice of reflections for refinement. *R*-factors based on *F*^2^ are statistically about twice as large as those based on *F*, and *R*- factors based on ALL data will be even larger.

### Fractional atomic coordinates and isotropic or equivalent isotropic displacement parameters (Å^2^)

**Table:**

	*x*	*y*	*z*	*U*_iso_*/*U*_eq_	
N1	−0.02089 (6)	0.95185 (13)	0.62956 (8)	0.0207 (3)	
N2	0.05790 (6)	0.82777 (12)	0.60540 (8)	0.0205 (3)	
N3	−0.02269 (6)	0.69671 (12)	0.62052 (8)	0.0215 (3)	
N4	0.05746 (6)	1.07016 (13)	0.61287 (9)	0.0230 (3)	
H4	0.0395 (8)	1.1562 (13)	0.6156 (13)	0.034*	
N5	0.05373 (6)	0.58500 (13)	0.59451 (9)	0.0232 (3)	
H5	0.0322 (7)	0.4997 (13)	0.5904 (13)	0.035*	
N6	−0.09379 (6)	0.82321 (14)	0.65212 (9)	0.0247 (3)	
H6	−0.1074 (8)	0.9110 (13)	0.6648 (13)	0.037*	
C1	0.03017 (7)	0.94590 (15)	0.61581 (9)	0.0193 (3)	
C2	0.02848 (7)	0.70791 (15)	0.60707 (9)	0.0198 (3)	
C3	−0.04387 (7)	0.82330 (15)	0.63353 (9)	0.0198 (3)	
C4	0.11497 (7)	1.06968 (16)	0.60499 (10)	0.0243 (3)	
H4A	0.1266	1.1685	0.5981	0.029*	
H4B	0.1097	1.0161	0.5472	0.029*	
C5	0.16599 (7)	1.00236 (16)	0.69218 (11)	0.0249 (3)	
C6	0.26088 (9)	0.8795 (3)	0.74867 (15)	0.0550 (6)	
H6A	0.2837	0.9527	0.7956	0.083*	
H6B	0.2881	0.8356	0.7243	0.083*	
H6C	0.2463	0.8067	0.7790	0.083*	
C7	0.09765 (7)	0.58905 (16)	0.55491 (11)	0.0255 (3)	
H7A	0.1119	0.4912	0.5522	0.031*	
H7B	0.1340	0.6456	0.5974	0.031*	
C8	0.07052 (7)	0.65318 (15)	0.45528 (11)	0.0243 (3)	
C9	0.09384 (10)	0.7424 (2)	0.33368 (14)	0.0436 (5)	
H9A	0.0706	0.6681	0.2880	0.065*	
H9B	0.1297	0.7701	0.3233	0.065*	
H9C	0.0672	0.8253	0.3246	0.065*	
C10	−0.12386 (7)	0.69593 (17)	0.66106 (10)	0.0257 (3)	
H10A	−0.1432	0.7149	0.7044	0.031*	
H10B	−0.0923	0.6210	0.6907	0.031*	
C11	−0.17285 (7)	0.64147 (16)	0.56692 (10)	0.0234 (3)	
C12	−0.25262 (8)	0.47226 (18)	0.49934 (12)	0.0335 (4)	
H12A	−0.2835	0.5442	0.4632	0.050*	
H12B	−0.2724	0.3961	0.5188	0.050*	
H12C	−0.2345	0.4327	0.4591	0.050*	
O1	0.20846 (5)	0.94345 (14)	0.67039 (8)	0.0397 (3)	
O2	0.16898 (5)	1.00247 (12)	0.77126 (8)	0.0325 (3)	
O3	0.11481 (5)	0.68883 (13)	0.43052 (8)	0.0362 (3)	
O4	0.01591 (5)	0.66684 (10)	0.40391 (7)	0.0255 (2)	
O5	−0.20447 (5)	0.53693 (12)	0.58329 (7)	0.0282 (3)	
O6	−0.18196 (6)	0.68287 (12)	0.48864 (8)	0.0336 (3)	

### Atomic displacement parameters (Å^2^)

**Table:**

	*U*^11^	*U*^22^	*U*^33^	*U*^12^	*U*^13^	*U*^23^
N1	0.0204 (7)	0.0224 (6)	0.0187 (6)	0.0007 (5)	0.0080 (5)	−0.0003 (5)
N2	0.0225 (7)	0.0194 (6)	0.0188 (6)	0.0006 (5)	0.0085 (5)	0.0002 (5)
N3	0.0245 (7)	0.0217 (7)	0.0190 (6)	−0.0017 (5)	0.0103 (5)	−0.0005 (5)
N4	0.0217 (7)	0.0186 (7)	0.0272 (7)	0.0015 (5)	0.0094 (6)	0.0020 (5)
N5	0.0293 (7)	0.0189 (7)	0.0236 (6)	0.0018 (5)	0.0137 (6)	0.0004 (5)
N6	0.0241 (7)	0.0270 (7)	0.0255 (6)	−0.0025 (6)	0.0132 (6)	−0.0051 (5)
C1	0.0205 (8)	0.0209 (7)	0.0126 (6)	−0.0002 (6)	0.0037 (6)	0.0005 (5)
C2	0.0238 (8)	0.0206 (7)	0.0129 (6)	0.0004 (6)	0.0061 (6)	0.0003 (5)
C3	0.0201 (7)	0.0242 (8)	0.0127 (6)	−0.0006 (6)	0.0051 (6)	−0.0018 (5)
C4	0.0245 (8)	0.0252 (8)	0.0229 (7)	−0.0024 (6)	0.0099 (7)	0.0042 (6)
C5	0.0227 (8)	0.0286 (8)	0.0226 (7)	−0.0042 (6)	0.0093 (7)	−0.0005 (6)
C6	0.0332 (11)	0.0885 (17)	0.0407 (11)	0.0262 (11)	0.0137 (9)	0.0179 (11)
C7	0.0275 (8)	0.0248 (8)	0.0269 (8)	0.0048 (7)	0.0145 (7)	0.0000 (6)
C8	0.0315 (9)	0.0175 (8)	0.0285 (8)	0.0028 (6)	0.0174 (7)	−0.0018 (6)
C9	0.0574 (12)	0.0458 (11)	0.0437 (11)	0.0095 (10)	0.0371 (10)	0.0135 (9)
C10	0.0265 (8)	0.0320 (9)	0.0215 (7)	−0.0045 (7)	0.0130 (7)	−0.0016 (6)
C11	0.0243 (8)	0.0257 (8)	0.0235 (7)	0.0007 (6)	0.0135 (7)	−0.0013 (6)
C12	0.0309 (9)	0.0375 (10)	0.0277 (8)	−0.0100 (8)	0.0089 (7)	−0.0075 (7)
O1	0.0268 (6)	0.0666 (9)	0.0275 (6)	0.0153 (6)	0.0137 (5)	0.0104 (6)
O2	0.0323 (7)	0.0442 (7)	0.0191 (5)	0.0018 (5)	0.0095 (5)	0.0001 (5)
O3	0.0351 (7)	0.0434 (7)	0.0386 (7)	0.0067 (6)	0.0241 (6)	0.0092 (5)
O4	0.0300 (6)	0.0202 (6)	0.0261 (6)	0.0024 (5)	0.0122 (5)	0.0008 (4)
O5	0.0279 (6)	0.0334 (6)	0.0229 (5)	−0.0075 (5)	0.0109 (5)	−0.0018 (4)
O6	0.0390 (7)	0.0393 (7)	0.0218 (5)	−0.0073 (5)	0.0128 (5)	0.0007 (5)

### Geometric parameters (Å, °)

**Table:**

N1—C1	1.3387 (18)	C6—H6A	0.9800
N1—C3	1.3430 (18)	C6—H6B	0.9800
N2—C2	1.3383 (18)	C6—H6C	0.9800
N2—C1	1.3428 (18)	C7—C8	1.520 (2)
N3—C2	1.3417 (18)	C7—H7A	0.9900
N3—C3	1.3458 (18)	C7—H7B	0.9900
N4—C1	1.3519 (19)	C8—O4	1.2096 (19)
N4—C4	1.4430 (18)	C8—O3	1.3266 (18)
N4—H4	0.927 (9)	C9—O3	1.455 (2)
N5—C2	1.3603 (18)	C9—H9A	0.9800
N5—C7	1.4399 (17)	C9—H9B	0.9800
N5—H5	0.943 (9)	C9—H9C	0.9800
N6—C3	1.3545 (18)	C10—C11	1.512 (2)
N6—C10	1.4377 (19)	C10—H10A	0.9900
N6—H6	0.941 (9)	C10—H10B	0.9900
C4—C5	1.514 (2)	C11—O6	1.2052 (18)
C4—H4A	0.9900	C11—O5	1.3355 (18)
C4—H4B	0.9900	C12—O5	1.447 (2)
C5—O2	1.2021 (18)	C12—H12A	0.9800
C5—O1	1.3316 (19)	C12—H12B	0.9800
C6—O1	1.447 (2)	C12—H12C	0.9800
			
C1—N1—C3	113.31 (12)	H6B—C6—H6C	109.5
C2—N2—C1	113.58 (12)	N5—C7—C8	112.37 (13)
C2—N3—C3	112.87 (12)	N5—C7—H7A	109.1
C1—N4—C4	119.93 (12)	C8—C7—H7A	109.1
C1—N4—H4	120.7 (11)	N5—C7—H7B	109.1
C4—N4—H4	119.4 (11)	C8—C7—H7B	109.1
C2—N5—C7	119.85 (12)	H7A—C7—H7B	107.9
C2—N5—H5	117.8 (11)	O4—C8—O3	124.06 (14)
C7—N5—H5	118.6 (11)	O4—C8—C7	124.99 (13)
C3—N6—C10	123.59 (12)	O3—C8—C7	110.92 (13)
C3—N6—H6	117.9 (11)	O3—C9—H9A	109.5
C10—N6—H6	118.3 (11)	O3—C9—H9B	109.5
N1—C1—N2	126.46 (13)	H9A—C9—H9B	109.5
N1—C1—N4	117.61 (13)	O3—C9—H9C	109.5
N2—C1—N4	115.93 (12)	H9A—C9—H9C	109.5
N2—C2—N3	126.84 (13)	H9B—C9—H9C	109.5
N2—C2—N5	116.12 (12)	N6—C10—C11	113.58 (12)
N3—C2—N5	117.05 (13)	N6—C10—H10A	108.8
N1—C3—N3	126.83 (12)	C11—C10—H10A	108.8
N1—C3—N6	115.64 (12)	N6—C10—H10B	108.8
N3—C3—N6	117.53 (12)	C11—C10—H10B	108.8
N4—C4—C5	110.88 (11)	H10A—C10—H10B	107.7
N4—C4—H4A	109.5	O6—C11—O5	124.43 (14)
C5—C4—H4A	109.5	O6—C11—C10	126.17 (14)
N4—C4—H4B	109.5	O5—C11—C10	109.39 (12)
C5—C4—H4B	109.5	O5—C12—H12A	109.5
H4A—C4—H4B	108.1	O5—C12—H12B	109.5
O2—C5—O1	123.66 (15)	H12A—C12—H12B	109.5
O2—C5—C4	125.40 (14)	O5—C12—H12C	109.5
O1—C5—C4	110.93 (12)	H12A—C12—H12C	109.5
O1—C6—H6A	109.5	H12B—C12—H12C	109.5
O1—C6—H6B	109.5	C5—O1—C6	116.24 (13)
H6A—C6—H6B	109.5	C8—O3—C9	115.57 (14)
O1—C6—H6C	109.5	C11—O5—C12	115.80 (12)
H6A—C6—H6C	109.5		
			
C3—N1—C1—N2	0.7 (2)	C10—N6—C3—N3	1.7 (2)
C3—N1—C1—N4	−178.61 (12)	C1—N4—C4—C5	−63.99 (17)
C2—N2—C1—N1	1.9 (2)	N4—C4—C5—O2	−29.1 (2)
C2—N2—C1—N4	−178.83 (12)	N4—C4—C5—O1	151.76 (13)
C4—N4—C1—N1	175.63 (12)	C2—N5—C7—C8	−60.15 (18)
C4—N4—C1—N2	−3.74 (19)	N5—C7—C8—O4	−17.7 (2)
C1—N2—C2—N3	−2.1 (2)	N5—C7—C8—O3	164.11 (12)
C1—N2—C2—N5	178.47 (12)	C3—N6—C10—C11	−86.95 (18)
C3—N3—C2—N2	−0.3 (2)	N6—C10—C11—O6	10.5 (2)
C3—N3—C2—N5	179.17 (12)	N6—C10—C11—O5	−170.70 (12)
C7—N5—C2—N2	−17.1 (2)	O2—C5—O1—C6	−1.1 (2)
C7—N5—C2—N3	163.40 (13)	C4—C5—O1—C6	178.03 (16)
C1—N1—C3—N3	−3.6 (2)	O4—C8—O3—C9	−2.2 (2)
C1—N1—C3—N6	176.83 (12)	C7—C8—O3—C9	175.96 (13)
C2—N3—C3—N1	3.4 (2)	O6—C11—O5—C12	−0.2 (2)
C2—N3—C3—N6	−177.03 (12)	C10—C11—O5—C12	−179.05 (13)
C10—N6—C3—N1	−178.64 (13)		

### Hydrogen-bond geometry (Å, °)

**Table:**

*D*—H···*A*	*D*—H	H···*A*	*D*···*A*	*D*—H···*A*
N4—H4···O4^i^	0.93 (1)	2.07 (1)	2.9851 (16)	168 (2)
N5—H5···O4^ii^	0.94 (1)	1.97 (1)	2.9097 (16)	172 (2)
N6—H6···O2^iii^	0.94 (1)	2.29 (1)	3.0733 (17)	141 (1)
C9—H9C···N1^i^	0.98	2.62	3.546 (3)	158

Symmetry codes: (i) −*x*, −*y*+2, −*z*+1; (ii) −*x*, −*y*+1, −*z*+1; (iii) −*x*, *y*, −*z*+3/2.
